# Primary malignant pericardial mesothelioma - a rare cause of pericardial effusion and consecutive constrictive pericarditis: a case report

**DOI:** 10.1186/1752-1947-0003-0000009256

**Published:** 2009-09-17

**Authors:** Thomas Butz, Lothar Faber, Christoph Langer, Jan Körfer, Oliver Lindner, Andrea Tannapfel, Klaus-Michael Müller, Axel Meissner, Gunnar Plehn, Hans-Joachim Trappe, Dieter Horstkotte, Cornelia Piper

**Affiliations:** 1 Department of Cardiology, Heart and Diabetes Center North Rhine-Westphalia, Ruhr-University Bochum, D-32545 Bad Oeynhausen, Germany; 2 Department of Cardiology and Angiology (Medizinische Klinik II), Marienhospital Herne, Ruhr-University Bochum, D-44627 Herne, Germany; 3 Institute of Radiology, Nuclear Medicine and Molecular Imaging, Heart and Diabetes Center North Rhine-Westphalia, Ruhr University Bochum, Bad Oeynhausen, Germany; 4 Institute of Pathology, Ruhr-University Bochum, Berufsgenossenschaftliche Universitätsklinik Bergmannsheil (Deutsches Mesotheliomregister), D-44789 Bochum, Germany

## Abstract

**Introduction:**

Primary malignant pericardial mesothelioma is a very rare pericardial tumor of unknown etiology.

**Case presentation:**

A 61-year-old Caucasian woman was admitted to our hospital complaining of exertional dyspnea due to a large pericardial effusion. Intrapericardial fluid volume declined after repeated pericardiocentesis, but the patient progressively developed a hemodynamically relevant pericardial constriction. Pericardiectomy revealed a pericardial mesothelioma. Subsequently, four cycles of chemotherapy (dosage according to recently published trials) were administered. The patient remained asymptomatic, and there was no recurrence of the tumor after three years.

**Conclusion:**

Pericardial mesothelioma should be considered and managed appropriately in non-responders to pericardiocentesis, and in patients who develop constrictive pericarditis late in their clinical course.

## Introduction

Primary malignant pericardial mesothelioma is a very rare pericardial tumor of unknown etiology.

## Case presentation

A 61-year-old Caucasian woman was admitted to our hospital complaining of exertional dyspnea (NYHA III) and chest pain. Transthoracic echocardiography demonstrated a large pericardial effusion. Pericardiocentesis revealed 1500 ml of an acellular, sterile pericardial effusion and symptoms were markedly relieved.

**Figure 1:**
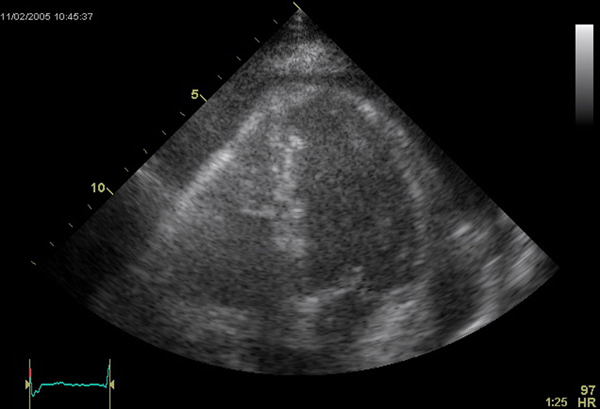
**Transthoracic echocardiography (apical 4-chamber view) demonstrating a large pericardial effusion and a thickened pericardium of the free wall of the right ventricle (see SD1 and SD2)**.

**Figure 2:**
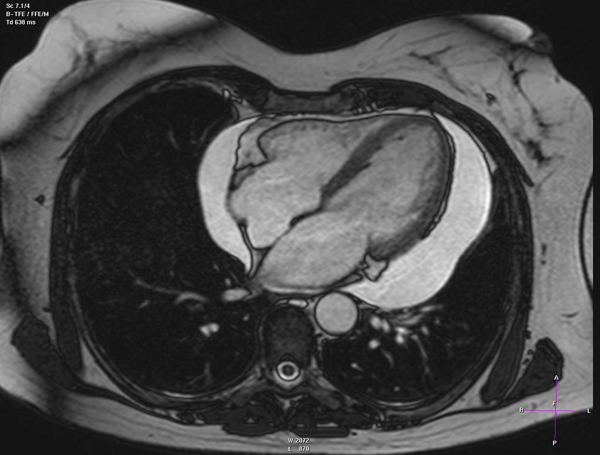
**Magnetic resonance imaging (4-chamber view, turbo field echo [TFE]) confirmed the extended pericardial effusion without signs of cardiac tamponade, and a slightly thickened pericardium (see SD3 and SD4)**.

The patient was re-admitted three months later, and transthoracic echocardiography showed a recurrent large pericardial effusion with partly organized fibrinous structures inside the effusion. There were no signs of cardiac tamponade, but there was a thickened right ventricular pericardium (Figure 1,SD1 and SD2). Magnetic resonance imaging (MRI) confirmed the pericardial effusion, and the slightly thickened pericardium (Figure 2,SD3 and SD4).

**Figure 3:**
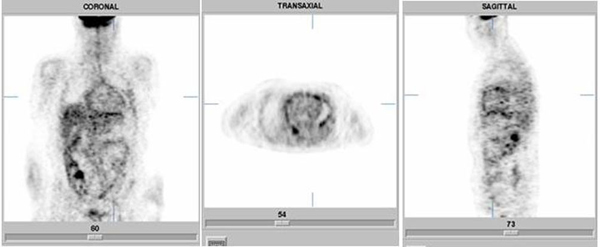
**F-18 fluorodeoxyglucose positron emission tomography/computed tomography (FDG-PET/CT) scan demonstrating an intrapericardial accumulation of the tracer (Siemens ECAT HR+)**.

An F-18 fluorodeoxyglucose positron emission tomography/computed tomography (FDG-PET/CT) scan demonstrated an intrapericardial accumulation of the tracer, indicating a local infection or a tumor (Figure 3) [1].

**Figure 4:**
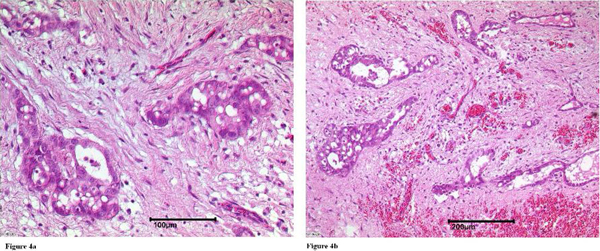
**Histological examination revealed diffuse infiltration of the pericardium by epithelioid cells due to the primary malignant pericardial mesothelioma (**a**: 100 μm, ** b**: 200 μm)**.

The patient's level of intrapericardial fluid declined after repeated pericardiocentesis, and cytology of the pericardial fluid revealed signs of chronic infection, but no malignant mesothelial cells. Subsequently, the patient developed a hemodynamically relevant pericardial constriction (SD5). Therefore, a partial pericardiectomy was performed, and histological examination (Figures 4a and 4b) revealed a primary malignant pericardial mesothelioma (PMPM). This finding initiated additional subtotal pericardiectomy with resection of as much pericardium as possible. The inspection of the epicardium by the surgeon showed a pericardial thickness of 10 mm and a white-colored spot of the pericardium at the right ventricle. There was no indication of tumor spread to adjacent structures, and there was no tumor on the epicardial site.

This was considered to be a PMPM because no signs of a pleural mesothelioma were found. Despite the above-mentioned findings of the magnetic resonance imaging (MRI) scan of the chest, FDG-PET, echocardiography and pericardiocentesis, we suspected PMPM but could not definitively declare a preoperative diagnosis of PMPM.

Subsequently, four cycles of chemotherapy with pemetrexed and cisplatin (four cycles in four months - dosage according to recently published trials) were administered, and remission was achieved [2]-[5]. The patient remained asymptomatic, and there was no recurrence of the tumor during the next three years.

## Discussion

Diagnosis of pericardial diseases can be challenging and often requires a multimodal imaging approach including echocardiography, MRI, CT and FDG-PET scans [6,7]. The majority of reported pericardial tumors are metastatic in nature and indicate a poor prognosis. Primary tumors of the pericardium are extremely rare, and PMPM is a very rare pericardial tumor of unknown etiology [8]-[10]. So far, about 350 cases have been reported in the literature, and in an epidemiological survey, the annual incidence of PMPM was reported to be one in 40 million (incidence 0.0022%). PMPM is characterized by atypical solid growth of the mesothelium with formation of atypical cavities surrounded by fibrous stroma.

There is some recent evidence that asbestos may have a harmful effect on pericardial serosa. However, there has not yet been any definite proven association between asbestos exposure and pericardial disease [2,8]-[10]. Interestingly, our patient had a history of asbestos exposure at work (she worked in a school building).

PMPM is often discovered late during a patient's clinical course or at autopsy. Frequent clinical diagnoses refer mainly to acute pericarditis, constrictive pericarditis, and cardiac tamponade and sometimes to various types of coronary heart disease.

Surgical resection remains the main treatment modality in PMPM. The prognosis of this disease remains extremely poor due to its late presentation, inability of complete tumor eradication by surgery and the poor response of PMPM to radiotherapy or chemotherapy. A median survival time from the onset of symptoms is six months [8]-[10]. Recently, newer chemotherapeutic regimens after complete excision of the tumor have shown prolonged survival times [2]-[5].

## Conclusion

PMPM should be considered and managed appropriately in non-responders to pericardiocentesis or pericardial window for treatment of pericardial effusion or tamponade, and in patients who develop constrictive pericarditis late in their clinical course.

## Abbreviations

CT: computer tomography; FDG: 2-fluoro-2-deoxy-d-glucose; FDG-PET: F-18 fluorodeoxyglucose positron emission tomography; MRI: magnetic resonance imaging; PMPM: primary malignant pericardial mesothelioma.

## Consent

Written informed consent was obtained from the patient for publication of this case report and any accompanying images. A copy of the written consent is available for review by the Editor-in-Chief of this journal.

## Competing interests

The authors declare that they have no competing interests.

## Authors' contributions

TB, LF, CL, AM, GP, HJT, DH and CP analyzed and interpreted the patient data regarding the cardiologic disease, therapy and the echocardiographic diagnostic. TB was a major contributor in writing the manuscript. JK analyzed and interpreted the magnetic resonance imaging; OL analyzed and interpreted the FDG-PET. AT and KMM performed the histological examination of the tumor. All authors read and approved the final manuscript.

## Supplementary Files

Movie 1 in 1. Transthoracic echocardiography demonstrated the recurrence of a large pericardial effusion and a thickened pericardium in the area of the right ventricle.

Movie 2 in 2. Transthoracic echocardiography (subcostal view) demonstrating a pericardial effusion and a markedly thickened pericardium.

Movie 3 in 3. Magnetic resonance imaging (MRI) confirmed the extended pericardial effusion without signs of cardiac tamponade, and a slightly thickened pericardium.

Movie 4 in 4. Magnetic resonance imaging (MRI) confirmed the extended pericardial effusion without signs of cardiac tamponade, and a slightly thickened pericardium.

Movie 5 in 5. Transthoracic echocardiography (subcostal view) demonstrating a markedly thickened pericardium and partly organised, fibrinous structures in the effusion.

## Supplementary Material

Additional file 1 Click here for file

Additional file 2 Click here for file

Additional file 3 Click here for file

Additional file 4 Click here for file

Additional file 5 Click here for file
